# Electrocardiographic Abnormalities and Treatment with Benznidazole among Children with Chronic Infection by *Trypanosoma cruzi*: A Retrospective Cohort Study

**DOI:** 10.1371/journal.pntd.0004651

**Published:** 2016-05-09

**Authors:** Lisandro D. Colantonio, Nilda Prado, Elsa L. Segura, Sergio Sosa-Estani

**Affiliations:** 1 Department of Epidemiology, School of Public Health, University of Alabama at Birmingham, Birmingham, Alabama, United States of America; 2 Department of Public Health, School of Medicine, University of Buenos Aires, Buenos Aires, Argentina; 3 National Institute of Parasitology “Dr. Mario Fatala Chaben”-CONICET-ANLIS, Ministry of Health, Buenos Aires, Argentina; Institute of Tropical Medicine, BELGIUM

## Abstract

**Background:**

Chronic infection by *Trypanosoma cruzi* could cause heart conduction disturbances. We sought to analyze electrocardiographic abnormalities among children with chronic *T*. *cruzi* infection with and without trypanocidal treatment with benznidazole.

**Methodology/Principal Findings:**

We studied 111 children 6–16 years of age with asymptomatic chronic *T*. *cruzi* infection who were recruited in 1991–1992 in Salta, Argentina. Most children were randomly assigned to benznidazole 5 mg/Kg/day (n = 47) or matching placebo (n = 48) for 60 days. Remaining children (n = 16) received treatment with benznidazole 5 mg/Kg/day open-label. Electrocardiograms were obtained at baseline and in 1995–1996, 1998, 2000 and 2005, and were analyzed using the Buenos Aires method. Among the 94 children with an electrocardiogram at baseline, 8 (8.5%) had electrocardiographic abnormalities, including 4 (4.7%) children with right bundle branch block. Proportion of abnormal electrocardiograms in the full population (n = 111) remained constant over time (media follow-up 8.6 years). Multivariable adjusted prevalence ratios (95% confidence interval [95%CI]) for electrocardiographic abnormalities in 1995–1996, 1998, 2000 and 2005 comparing children treated with benznidazole versus those not treated were 2.76 (0.66, 11.60), 2.33 (0.44, 12.31), 3.06 (0.48, 19.56), and 1.94 (0.33, 11.25), respectively. Among the 86 children with a normal electrocardiogram at baseline, 16 (18.6%) developed electrocardiographic abnormalities during follow-up. The multivariable adjusted hazard ratio for incident electrocardiographic abnormalities comparing children treated with benznidazole versus those not treated was 0.68 (95%CI: 0.25, 1.88).

**Conclusions/Significance:**

Electrocardiographic abnormalities are frequent among children with chronic *T*. *cruzi* infection. Treatment with benznidazole for 60 days may not be associated with less electrocardiographic abnormalities.

## Author Summary

There are few data available on the natural history of electrocardiographic abnormalities among children with chronic *Trypanosoma cruzi* infection. Also, few studies analyzed the effect of benznidazole to prevent electrocardiographic abnormalities in this population. In the current study, electrocardiographic abnormalities were frequent among children with chronic *T*. *cruzi* infection. Results from the current study also suggest that treatment with benznidazole may not be associated with less electrocardiographic abnormalities. The current study highlights the need of further research to prevent cardiovascular manifestations associated with chronic *T*. *cruzi* infection.

## Introduction

Chagas’ disease is a chronic condition characterized by cardiovascular, digestive and neurologic manifestations, which is caused by a vector borne parasitic infection (*Trypanosoma cruzi)* endemic in Latin America [1]. Chagas’ disease is an important cause of premature death, disability, reduced quality of life and high costs for health systems in endemic countries [1, 2]. Emigration from Latin America (mainly to US, Canada, Europe and Australia) as well as alternative routes of transmission (i.e., vertical or through blood transfusion) have transformed Chagas’ disease in a major global threat [3–6]. Every year, Chagas’ disease is responsible for 806,170 disability-adjusted life-years lost and US$ 627.46 million in direct healthcare costs worldwide, with more than 14% of these costs emanating from non-endemic countries [7].

Most individuals with Chagas’ disease have chagasic cardiomyopathy [1, 8, 9]. Chagasic cardiomyopathy usually appears in the adulthood, after 10 to 20 years of chronic infection by *T*. *cruzi* [1]. However, early stages of chagasic cardiomyopathy can also be detected among children or adolescents [10]. Chagasic cardiomyopathy is commonly preceded by heart conduction disturbances, which can be detected through electrocardiography [9, 11–13]. Electrocardiographic abnormalities are considered an important marker of chagasic cardiomyopathy severity and progression [9, 11].

Benznidazole is effective to induce parasite clearance [14–17] and is recommended for treatment of acute, congenital and reactivated *T*. *cruzi* infection as well as among children with chronic infection [18, 19]. Evidence from animal models suggest that treatment with benznidazole could prevent or control chagasic cardiomyopathy [20], although results from observational studies have been controversial [16]. Treatment with benznidazole for 60 days was not effective to prevent clinical progression in adults with chagasic cardiomyopathy (mean age 55 years) in a large randomized clinical trial (Benznidazole Evaluation for Interrupting Trypanosomiasis, BENEFIT, NCT00123916) [21]. These results support current guidelines which do not recommend treatment with benznidazole among individuals with chronic *T*. *cruzi* infection 50 years of age or older or with advanced cardiomyopathy [18, 22].

Few studies analyzed the characteristics and natural history of electrocardiographic abnormalities among children with chronic *T*. *cruzi* infection and the effect associated with treatment with benznidazole [16, 19]. The main objective of the present study was to investigate the presence of electrocardiographic abnormalities in a cohort of children with chronic *T*. *cruzi* infection, some of whom received treatment with benznidazole. We hypothesized that electrocardiographic abnormalities will be frequent among children with chronic *T*. *cruzi* infection and less common among those treated with benznidazole versus those not treated.

## Methods

### Population and procedures

We conducted a retrospective cohort study using data collected during a double-blind randomized controlled clinical trial with extended follow-up. The clinical trial was conducted in Salta province, Argentina from 1991 through 1996 and was aimed at investigating the parasite clearance and safety associated with treatment with benznidazole among children 6 to 12 years of age with asymptomatic chronic infection by *T*. *cruzi* [15]. The region where the study was conducted had continuous surveillance for *T*. *cruzi* vectors by sanitary agents since 1982, and the possibility of reinfection after treatment was considered low. During enrolment in 1991–1992, children attending local elementary schools were screened by history, physical examination and 3 serology tests for *T*. *cruzi* using different techniques: indirect hemagglutination inhibition, indirect immunofluorescence assay, and enzyme-linked immunosorbent assay. Of relevance to the current analysis, information on age, sex, body weight and place of residence was collected. Children were considered eligible for the clinical trial if they had chronic infection by *T*. *cruzi*, defined by at least 2 positive serology tests using different techniques. Exclusion criteria were presence of any acute infection or chronic condition, or unstable residence. Children with Chagas’ disease, defined by the presence of cardiovascular, digestive or neurologic symptoms, were excluded from the study.

Children included in the clinical trial were matched by age and place of residence, and randomly assigned to benznidazole 5 mg/Kg/day (benznidazole group, n = 55) or placebo (placebo group, n = 51) for 60 days. Participants were followed for 48 months through 1995–1996. At the end of the clinical trial, all participants in the placebo group were offered treatment with benznidazole if follow-up for adverse events was considered feasible. A total of 18 children in the placebo group completed a properly documented treatment with benznidazole open-label in 1997, following the same treatment protocol as in the clinical trial. No treatment with benznidazole was documented among the remaining 33 children assigned to placebo. An enzyme-linked immunosorbent assay using a flagellar calcium-binding protein F29 (F29 ELISA) and xenodiagnosis were performed in children included in the clinical trial in 2005.

A cohort of 19 children with asymptomatic chronic infection by *T*. *cruzi* who completed the study anamnesis, physical examination and serology tests but were not included in the clinical trial received treated with benznidazole open-label 5 mg/Kg/day for 60 days in 1991–1992 (benznidazole cohort). Reasons for exclusion from the clinical trial included age <6 or >12 years, abnormal laboratory parameters (leukocytosis or anemia), intestinal parasitosis, and unstable residence.

Electrocardiograms were obtained from children enrolled in the clinical trial and in the benznidazole cohort in 1991–1992, 1995–1996, 1998, 2000 and 2005. For the present analysis, we included children enrolled in the clinical trial or in the benznidazole cohort who had at least 1 valid electrocardiogram to assess electrocardiographic abnormalities.

### Ethics statement

Written informed consent to participate in the study was obtained from legal guardians before assessment for enrollment and once again before randomization among those included in the clinical trial. Written informed consent to participate in subsequent examinations (i.e., in 1998, 2000 and 2005) was obtained from the participant or the legal guardian, according the participant’s age. The protocol was reviewed and approved by the Institutional Review Board of the “Instituto Nacional de Chagas Dr. Mario Fatala Chaben”, Buenos Aires, Argentina before the study initiation. Study procedures were conducted in accordance with the principles stated in the Declaration of Helsinki.

### Variables included in the analysis

All electrocardiograms were analyzed by the same cardiologist (NP) using the Buenos Aires method [23]. The Buenos Aires method is an electrocardiographic recording and reading guide designed for epidemiological studies on Chagas’ disease. This method analyzes 5 electrocardiographic measurements and 48 items classified into 9 diagnostic categories: overall assessment, rhythm, supraventricular arrhythmias, ventricular arrhythmias, atrioventricular conduction disturbances, ventricular conduction defects, abnormal initial QRS complex, primary ST-T wave changes, and miscellaneous. The inter-rater agreement to identify electrocardiographic abnormalities using the Buenos Aires method is substantial (kappa statistic: 0.66, standard error: 0.02) [23]. Electrocardiograms with missing date or coded as not evaluable by the cardiologist were excluded from the current analysis. Electrocardiographic abnormalities were defined by any abnormal finding identified using the Buenos Aires method.

Potential confounders assessed at baseline which were used for statistical adjustment in the present analysis include age, gender, body weight and rural residence.

### Statistical analysis

Baseline characteristics of participants included in the current analysis who were randomly assigned to benznidazole and placebo, separately, and those in the benznidazole cohort are reported using median and 25^th^-75^th^ percentiles for continuous variables, and percentage for binary variables. Differences between groups were analyzed using Kruskal-Wallis or Fisher’s exact tests, as appropriated.

We calculated the proportion of electrocardiograms with electrocardiographic abnormalities in each assessment period (i.e., 1991–1992, 1995–1996, 1998, 2000 and 2005), separately. Some participants have more than 1 electrocardiogram recorded in the same period. Therefore, we used a mixed effect model with random intercept to estimate appropriated 95% confidence intervals (CI) taking into account repeated measurements from a same participant. We used the last observation carried forward method for the main analysis because some participants did not have an electrocardiogram in each assessment period. The regression model was fit using the maximum likelihood method with a binomial distribution and log link, and including the assessment periods as dummy variables with 1991–1992 as the reference. We used least-squares means to estimate the proportion and 95% CI for electrocardiographic abnormalities in each period. We tested for a trend in the proportion of electrocardiograms with electrocardiographic abnormalities over time by analyzing the assessment period as an ordinal variable.

We used a panel analysis and Poisson regression models with robust variance to analyze the association between treatment with benznidazole and electrocardiographic abnormalities. We used Poisson regression models because log-binomial regression models including multivariable adjustment did not converge. Model 1 included terms for each assessment period using dummy variables as described above and an indicator variable for treatment with benznidazole. Model 2 included variables in Model 1 plus age, gender, body weight and rural residence. All models included interactions between each assessment period and treatment with benznidazole to estimate the effect modification associated with benznidazole after baseline [24]. Prevalence ratios and 95% CI for electrocardiographic abnormalities were estimated after exponentiation of coefficients for the interactions between the assessment periods and treatment with benznidazole. These prevalence ratios represent the relative effect of benznidazole on electrocardiographic abnormalities after adjusting for baseline differences in the proportion of electrocardiograms with abnormalities between children treated and not treated with benznidazole.

For our main analysis, we considered that children randomly assigned to benznidazole in the clinical trial and those in the benznidazole cohort were treated with this medication (intention-to-treat analysis). Several sensitivity analyses of the association between treatment with benznidazole and electrocardiographic abnormalities were conducted. First, we repeated the analysis without using the last observation carried forward method. Second, we conducted a per-protocol analysis, considering children who did not complete 30 days of treatment with benznidazole as not treated, and children who received treatment with benznidazole in 1997 as treated in 1998, 2000 and 2005. We used 30 days to determine whether children were treated as prior studies have shown that a treatment with benznidazole shorter than 60 days can be effective to induce *T*. *cruzi* clearance [25, 26]. Finally, we repeated the analysis limited to children enrolled in the randomized controlled clinical trial.

We conducted analyses limited to children without electrocardiographic abnormalities at baseline. Baseline characteristics of participants in these analyses as well as the characteristics of incident electrocardiographic abnormalities and the proportion of electrocardiograms with abnormalities over time were calculated as described above. We used a Weibull regression model (accelerated failure time) to conduct an interval-censoring analysis of the association between treatment with benznidazole and incident electrocardiographic abnormalities among children with a normal electrocardiogram at baseline [27]. Hazard ratios and 95% CI for incident electrocardiographic abnormalities associated with treatment with benznidazole were calculated as described by Collett [28]. In addition to a crude model, a multivariable adjusted model was fit, including adjustment for age, gender, body weight and rural residence. Finally, we analyzed F29 ELISA and xenodiagnosis results in 2005 among children treated with benznidazole in the clinical trial who had incident electrocardiographic abnormalities.

All statistical analyses were performed using SAS v. 9.4 (SAS Institute Inc., Cary, NC). All tests were 2-sided and used a level of significance alpha <0.05.

## Results

**Fig 1:**
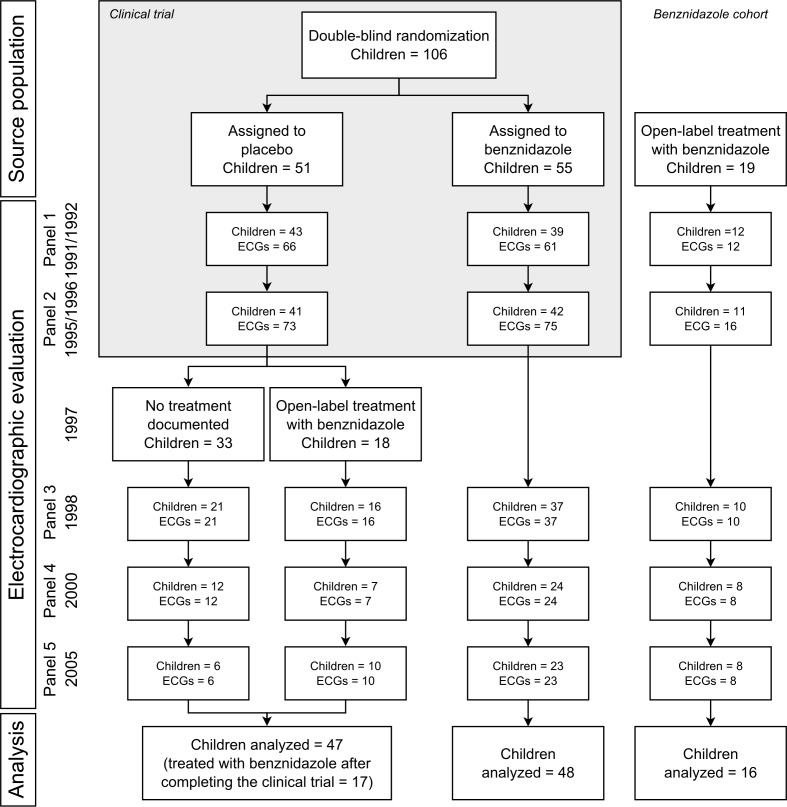
Flowchart of participants included in the analysis. ECG: electrocardiogram.

Children in the benznidazole cohort (n = 19) were similar to participants enrolled in the clinical trial (n = 106) regarding gender (47.4% vs 52.8% females, Fisher’s exact test p-value: 0.80) and place of residence (57.9% vs 45.3% rural residents, p-value: 0.33), but were older (median age in years [25^th^-75^th^ percentiles]: 10 [9–14] vs 10 [8–11], Wilcoxon rank-sum test p-value: 0.05). Between 1991 and 2005, 500 electrocardiograms were obtained from this population. We excluded electrocardiograms with missing date (n = 1) and those coded as not evaluable by the cardiologist (n = 14). After these exclusions, 111 children had at least 1 valid electrocardiogram during the study period (485 electrocardiograms in total) and were included in the current analysis. The distribution of children and electrocardiograms included in the analysis is shown in **Fig 1**. The number of children who participated at follow-up examinations and had an electrocardiogram was reduced over time, mainly because migration.

Children included in the analysis who were assigned to the benznidazole (n = 48) and placebo (n = 47) groups of the clinical trial, separately, and those in the benznidazole cohort (n = 16) were similar in age, body weight, gender and rural residence (**Table 1**, top panel). However, children in the benznidazole cohort had less electrocardiograms during follow-up as compared to those enrolled in the clinical trial. Median follow-up (25^th^-75^th^ percentile) was 8.6 (7.1–14.1) years.

**Table 1:** Baseline characteristics of children included in the analysis (n = 111) and those without electrocardiographic abnormalities at baseline (n = 86).

	Double-blind randomized clinical trial			
	Assigned to placebo	Assigned to benznidazole	Benznidazole cohort	p-value
*Children included in the analysis*, *n*	*47*	*48*	*16*	
	*n (%)*			
Female	23 (49)	25 (52)	8 (50)	0.97
Rural residence	22 (49)	20 (42)	10 (63)	0.34
	*Median (25*^*th*^*-75*^*th*^ *percentiles)*			
Age, years	10 (9, 11)	10 (8, 12)	10 (9, 14)	0.37
Weight, Kg	30 (24, 38)	31 (26, 38)	34 (26, 47)	0.31
Number of electrocardiograms	5 (4, 6)	5 (4, 6)	4 (2, 4)	0.03
Follow-up, years	7.6 (7.1, 14.1)	8.6 (7.1, 14.1)	11.3 (7.0, 14.0)	0.43
*Children without electrocardiographic abnormalities at baseline*, *n*	*38*	*37*	*11*	
	*n (%)*			
Female	20 (53)	17 (46)	7 (64)	0.63
Rural residence	16 (42)	17 (46)	7 (64)	0.50
	*Median (25*^*th*^*-75*^*th*^ *percentiles)*			
Age, years	10 (8, 11)	10 (8, 12)	10 (9, 14)	0.38
Weight, Kg	30 (24, 37)	31 (25, 38)	33 (25, 49)	0.33
Number of electrocardiograms	5 (4, 6)	5 (4, 6)	4 (3, 4)	0.04
Follow-up, years	8.1 (7.1, 14.1)	13.0 (7.1, 14.1)	8.6 (6.9, 14.0)	0.44

Note: Variables reported have no missing data.

**Fig 2:**
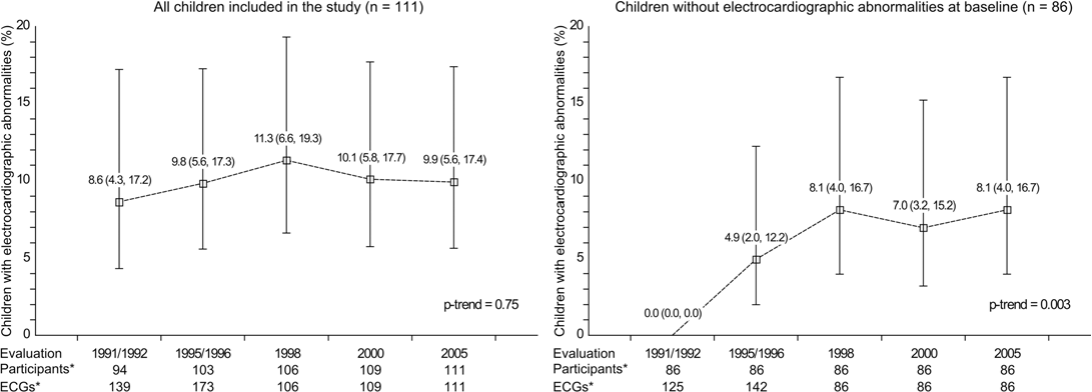
Proportion of electrocardiograms with abnormalities by assessment period among all children included in the analysis (n = 111) and children without electrocardiographic abnormalities at baseline (n = 86). Labels represent proportion (95% confidence intervals). * Using the last observation carried forward method. ECG: electrocardiogram.

A total of 94 children had an electrocardiogram in 1991–1992, including 8 (8.5%) children with electrocardiographic abnormalities (**Table 2**). Most common electrocardiographic abnormalities included rR’ or R wave in V1 (i.e., right bundle branch block) and left anterior fascicular block. Proportion of electrocardiograms with electrocardiographic abnormalities reminded relatively stable across assessment periods, ranging from 8.6% (95% CI: 4.3%-16.7%) in 1991–1992 to 11.3% (95% CI: 6.5%-18.9%) in 1998 (**Fig 2**, left panel).

**Table 2:** Description of baseline electrocardiograms in 1991–1992 according the Buenos Aires method (n = 94).

Buenos Aires method	All children (n = 94)
**Electrocardiographic measurements**	*Median / 25*^*th*^ *and 75*^*th*^ *percentiles / minimum and maximum*
Heart rate, beats per minute	86 / 77, 96 / 63, 123
PR interval, seconds/100	0.12 / 0.12, 0.14 / 0.10, 0.20
QRS duration, seconds/100	0.07 / 0.06, 0.08 / 0.05, 0.08
QRS axis, degrees	60 / 45, 60 / -45, 150
Ventricular extrasystoles, n	0 / 0, 0 / 0, 0
Electrocardiographic items*	*n (%)*
Overall assessment	
Normal	86 (91)
Abnormal	8 (9)
Rhythm	
Sinus	94 (100)
Supraventricular arrhythmias	
None	92 (98)
Supraventricular extrasystoles	2 (2)
Ventricular arrhythmias	
None	94 (100)
Atrioventricular conduction disturbances	
None	94 (100)
Ventricular conduction defects	
None	86 (92)
rR’ or R in V1 (i.e., right bundle branch block)	4 (4)
Left anterior fascicular block	3 (3)
Noncodifiable	1 (1)
Abnormal initial QRS complex	
None	92 (98)
Noncodifiable	2 (2)
Primary ST-T wave changes	
None	93 (99)
Noncodifiable	1 (1)
Miscellaneous	
None	93 (99)
Right ventricular hypertrophy	1 (1)

* Only electrocardiographic items with non-zero values are displayed for simplicity.

The prevalence of electrocardiograms with abnormalities was higher among children treated with benznidazole compared with those not treated in all assessment periods following the baseline evaluation (**Table 3**). Prevalence ratios for electrocardiographic abnormalities associated with treatment with benznidazole were not statistically significant in any post-baseline assessment. Results were similar in sensitivity analyses.

**Table 3:** Association between treatment with benznidazole and electrocardiographic abnormalities according the Buenos Aires method.

	Panel 1	Panel 2	Panel 3	Panel 4	Panel 5	
	1991–1992	1995–1996	1998	2000	2005	p-value
**Main analysis (n = 111)**						
*Proportion of electrocardiograms with electrocardiographic abnormalities*, *% (95% CI)*						
Treatment with benznidazole	6.8% (2.2%, 20.9%)	12.4% (6.1%, 25.1%)	13.3% (7.0%, 25.5%)	12.9% (6.7%, 24.7%)	10.9% (5.4%, 22.1%)	-
No treatment with benznidazole	10.6% (4.4%, 25.5%)	6.6% (2.8%, 15.2%)	8.7% (3.4%, 22.2%)	6.4% (2.1%, 19.1%)	8.5% (3.3%, 21.8%)	-
*Treatment with benznidazole vs no treatment*, *PR (95% CI)*						
Model 1	-	2.91 (0.74, 11.46)	2.37 (0.46, 12.25)	3.13 (0.50, 19.53)	1.99 (0.35, 11.35)	0.55
Model 2	-	2.76 (0.66, 11.60)	2.33 (0.44, 12.31)	3.06 (0.48, 19.56)	1.94 (0.33, 11.25)	0.63
***Sensitivity analyses***						
**Analysis without the last observation carried forward method (n = 111)**						
*Proportion of electrocardiograms with electrocardiographic abnormalities*, *% (95% CI)*						
Treatment with benznidazole	6.8% (2.2%, 20.9%)	13.2% (6.5%, 26.8%)	17.0% (9.0%, 32.1%)	21.9% (11.3%, 42.2%)	12.9% (5.1%, 32.3%)	-
No treatment with benznidazole	10.6% (4.4%, 25.5%)	6.8% (3.0%, 15.9%)	10.8% (4.3%, 27.4%)	5.3% (0.8%, 35.8%)	18.7% (6.7%, 52.3%)	-
*Treatment with benznidazole vs no treatment*, *PR (95% CI)*						
Model 1	-	2.98 (0.75, 11.79)	2.44 (0.49, 12.17)	6.44 (0.57, 72.83)	1.07 (0.18, 6.27)	0.27
Model 2	-	2.95 (0.70, 12.51)	2.38 (0.47, 12.11)	6.55 (0.57, 75.31)	0.90 (0.14, 5.53)	0.26
**Analysis per protocol (n = 111)**						
*Proportion of electrocardiograms with electrocardiographic abnormalities*, *% (95% CI)*						
Treatment with benznidazole	7.2% (2.4%, 22.0%)	13.0% (6.4%, 26.4%)	13.2% (7.4%, 23.5%)	13.0% (7.8%, 21.7%)*		-
No treatment with benznidazole	10.0% (4.1%, 24.1%)	6.2% (2.7%, 14.3%)	6.7% (1.7%, 25.6%)	1.7% (0.2%, 11.7%)*		-
*Treatment with benznidazole vs no treatment*, *PR (95% CI)*						
Model 1	-	2.37 (0.73, 7.69)	1.78 (0.47, 6.83)	8.65 (0.89, 84.31)*		0.21
Model 2	-	2.56 (0.77, 8.59)	1.86 (0.42, 8.21)	8.63 (0.82, 90.95)*		0.27
**Restricted to children enrolled in the randomized controlled clinical trial (n = 95)**						
*Proportion of electrocardiograms with electrocardiographic abnormalities*, *% (95% CI)*						
Treatment with benznidazole	6.6% (1.8%, 24.4%)	12.8% (5.9%, 27.6%)	15.2% (7.7%, 30.2%)	10.6% (4.6%, 24.4%)	8.3% (3.3%, 21.4%)	-
No treatment with benznidazole	10.6% (4.4%, 25.5%)	6.6% (2.8%, 15.2%)	8.7% (3.4%, 22.3%)	6.4% (2.1%, 19.2%)	8.5% (3.3%, 21.8%)	-
*Treatment with benznidazole vs no treatment*, *PR (95% CI)*						
Model 1	-	3.15 (0.65, 15.19)	2.83 (0.46, 17.26)	2.70 (0.37, 19.87)	1.58 (0.23, 10.75)	0.60
Model 2	-	2.92 (0.56, 15.15)	2.72 (0.44, 16.85)	2.59 (0.34, 19.54)	1.52 (0.22, 10.52)	0.68

Model 1 adjusts for the difference in the proportion of electrocardiograms with electrocardiographic abnormalities in 1991–1992 between children treated and not treated with benznidazole. Model 2 adjusts for covariates in Model 1 plus age, gender, rural residence and body weight.

* Electrocardiograms in panels 4 and 5 were combined to prevent cells with 0 cases

CI: confidence intervals; PR: prevalence ratio.

Baseline characteristics of the 86 children without electrocardiographic abnormalities in 1991–1992 are shown in **Table 1**, bottom panel. The proportion of electrocardiograms with electrocardiographic abnormalities increased over time in this population (**Fig 2**, right panel). A total of 16 (18.6%) children developed incident electrocardiographic abnormalities during follow-up, including 8 participants who received treatment with benznidazole in 1991–1992 (**Table 4**). Among those with incident electrocardiographic abnormalities, 4 children (3 children treated with benznidazole) developed rR’ or R in V1. The crude hazard ratio for incident electrocardiographic abnormalities comparing children treated with benznidazole versus those not treated was 0.74 (95% CI: 0.28–1.97, p-value: 0.54). After adjustment for age at baseline, gender, rural residence and body weight, the hazard ratio for incident electrocardiographic abnormalities associated with treatment with benznidazole was 0.68 (95% CI: 0.25–1.88, p-value: 0.46). All of the 5 children treated with benznidazole in the clinical trial who had incident electrocardiographic abnormalities and completed a F29 ELISA and xenodiagnosis test in 2005 had negative results for *T*. *cruzi* infection.

**Table 4:** Incident electrocardiographic abnormalities among children with a normal electrocardiogram at baseline (1991–1992) according the Buenos Aires method (n = 86).

	No treatment with benznidazole (n = 38)	Treatment with benznidazole (n = 48)
Electrocardiographic items	*n (%)*	*n (%)*
Overall assessment		
Abnormal	8 (21)	8 (17)
Rhythm		
Atrial ectopic	1 (3)	0 (0)
Supraventricular arrhythmias		
Supraventricular extrasystoles	1 (3)	0 (0)
Other atrial arrhythmias	0 (0)	1 (2)
Ventricular arrhythmias		
Simple ventricular extrasystoles	0 (0)	2 (4)
Atrioventricular conduction disturbances		
Ventricular pre-excitation	1 (3)	0 (0)
Ventricular conduction defects		
rR’ or R in V1 (including right bundle branch block)	1 (3)	3 (6)
Left anterior fascicular block	1 (3)	0 (0)
Left posterior fascicular block	0 (0)	1 (2)
Nonspecific intraventricular conduction defect	1 (3)	0 (0)
Abnormal initial QRS complex		
Present	1 (3)	0 (0)
Miscellaneous		
Right ventricular hypertrophy	1 (3)	1 (2)
P wave	1 (3)	0 (0)

Note: only items with abnormal findings are displayed for simplicity.

## Discussion

We analyzed the characteristics and frequency of electrocardiographic abnormalities among children with chronic *T*. *cruzi* infection and the effect associated with treatment with benznidazole. In our analysis, children with chronic *T*. *cruzi* infection frequently presented or developed electrocardiographic abnormalities. Most of these electrocardiographic abnormalities, including right bundle branch block, and left anterior fascicular block, are more common among individuals with chronic *T*. *cruzi* infection [29]. After statistical adjustment, treatment with benznidazole for 60 days was not associated with less electrocardiographic abnormalities as compared with no treatment over a median follow-up of 8.6 years. We found no evidence of treatment failure among children with incident electrocardiographic abnormalities who completed treatment with benznidazole at baseline. These results should be interpreted in the context of the few number of participants analyzed, the number of electrocardiographic abnormalities and the long term evolution of chagasic cardiomyopathy.

Treatment with benznidazole is currently recommended in acute, congenital and reactivated infection by *T*. *cruzi*, and among children with chronic infection [18, 19, 30, 31]. The recommendation to treat children with chronic *T*. *cruzi* infection is based on 2 clinical trials conducted in the 1990s which found that benznidazole is effective to induce *T*. *cruzi* clearance in this population [14, 15]. However, these studies have not shown that treatment with benznidazole can prevent heart conduction disturbances or chagasic cardiomyopathy. In 1991, de Andrade et al. carried out a clinical trial including 130 children 7 to 12 years of age with chronic *T*. *cruzi* infection who were randomly assigned 1:1 to treatment with benznidazole 7.5 mg/Kg/day or placebo for 60 days [14]. Over 3 years of follow-up, 1 child in the treatment group and 4 children in the control group developed a complete right bundle branch block (p-value: 0.36). None of the children showed evidence of chagasic cardiomyopathy after 6 years of follow-up in the extension study [32]. Using data from the other clinical trial conducted in the 1990s, we observed no difference in the presence of a broader spectrum of electrocardiographic abnormalities between children with chronic *T*. *cruzi* infection who were and were not treated with benznidazole. Results from these studies are important considering the few data available about the natural history of electrocardiographic abnormalities and the effect of treatment with benznidazole on heart conduction disturbances among children with chronic *T*. *cruzi* infection. Taken together, results from these studies suggest that treatment with benznidazole in the current scheme of 60 days may not prevent electrocardiographic abnormalities in this population.

Few studies have investigated the efficacy of benznidazole to prevent electrocardiographic abnormalities or Chagas’ heart disease progression among adults [16, 33, 34]. In the BENEFIT trial, treatment with benznidazole did not reduce the risk for clinical outcomes compared with placebo among 2,854 adults with chagasic’ cardiomyopathy. After 7 years of follow-up, the hazard ratio for death, cardiac arrest, insertion of a pacemaker or an implantable cardioverter–defibrillator, sustained ventricular tachycardia, cardiac transplantation, new heart failure, stroke, transient ischemic attack, or a thromboembolic event associated with benznidazole was 0.93 (95% CI: 0.81–1.07). These results support some current guidelines which do not recommend treatment with benznidazole among adults more than 50 years old with chronic *T*. *cruzi* infection given their lower rate of seroconversion and a higher risk for side effects compared with children [18, 19, 35]. Results from TRAENA (NCT02386358), another large randomized clinical trial designed to investigate the efficacy of benznidazole to prevent major cardiovascular outcomes among adults with Chagas’ disease, are expected to be published by the end of 2016 [36].

In our analysis, we found no evidence of persistent *T*. *cruzi* infection among children with incident electrocardiographic abnormalities who received treatment with benznidazole. However, conventional tests for *T*. *cruzi* infection (i.e., serology tests, xenodiagnosis and polymerase chain reaction [PCR]) have important limitations to determine a complete parasite elimination following treatment with benznidazole [37]. Specifically, serology tests may remain positive several years after treatment with benznidazole, and xenodiagnosis and PCR are negative in many individuals with chronic *T*. *cruzi* infection [19, 38]. Prior studies have shown a trypanocidal effect of benznidazole among individuals with chronic *T*. *cruzi* infection using serology tests, xenodiagnosis and PCR [14–17, 34]. However, some individuals may remain with persistent *T*. *cruzi* infection after treatment. In the BENEFIT trial, 53.3% of participants with a positive PCR test at baseline who received treatment with benznidazole had a positive PCR test after 5 years of follow-up [21]. Even a few number of *T*. *cruzi* specimens could be enough to maintain an autoimmune response with production of antibodies against nervous and cardiac muscle which could play an important role in the development and progression of heart conduction disturbances and chagasic cardiomyopathy [30, 33, 39]. This could have contributed to an apparent lack of efficacy of benznidazole to prevent electrocardiographic abnormalities or chagasic cardiomyopathy in prior studies. Future studies should focus on developing diagnostic methods with high sensitivity and specificity to detect persistent *T*. *cruzi* infection after treatment with trypanocidal drugs as well as to determine whether individuals with persistent *T*. *cruzi* infection would benefit from retreatment.

Most participants included in the present analysis were initially enrolled in a clinical trial and all procedures for data collection followed the same study protocol. Also, treatment with benznidazole was performed in accordance with current recommendations [18, 19, 35]. Despite these strengths, results from the present analysis should be interpreted in the context of potential limitations. Following young populations in low resource settings is challenging and some study participants were lost during follow-up, mainly because migration. Only few covariates were available for statistical adjustment for potential confounders, which is related to the fact that risk factors for chagasic cardiomyopathy among individuals with chronic *T*. *cruzi* infection remain largely unknown. Several factors may have contributed to attenuate a possible association between treatment with benznidazole and lower risk for electrocardiographic abnormalities in our study. Some children who received treatment with benznidazole may have remained with persistent *T*. *cruzi* infection. We cannot exclude the possibility of reinfection among children successfully treated with benznidazole, although this was considered unlikely. Also, some children who were analyzed as untreated in the present study may have received treatment with benznidazole during follow-up, which was not documented. Some electrocardiographic abnormalities observed in this analysis, including bundle branch block, may be unrelated with *T*. *cruzi* infection as they may also be detected among uninfected children [29, 40]. However, a prior study using the Buenos Aires method reported that electrocardiographic abnormalities, including bundle branch block and left anterior fascicular block, are more common among individuals with chronic *T*. *cruzi* infection compared with uninfected controls [29]. Because the small sample size, our analysis had low statistical power to exclude a clinically relevant effect to prevent electrocardiographic abnormalities associated with treatment with benznidazole. For example, in our main analysis, the lower bound of the 95% CI for the prevalence ratio in the second assessment period was 0.66. This means that we cannot exclude a reduction on the prevalence of electrocardiographic abnormalities associated with benznidazole as large as 44%. Finally, because the study was conducted in a restricted geographic area, results may not be generalizable to children with chronic *T*. *cruzi* infection from other regions.

Results from the present study should not be interpreted as indicative that children with chronic *T*. *cruzi* infection should not be treated with benznidazole. Treatment with benznidazole among children with chronic *T*. *cruzi* infection can contribute to induce *T*. *cruzi* clearance [14, 15], and reduce the number of incident cases by vertical transmission [41–43], blood transfusion and organ transplant [3, 31]. Treatment with benznidazole can also contribute to reduce the risk for a reactivation associated with immunodeficiency disorders or immunosuppression, and reaching seroconversion could be beneficial for the wellbeing of children and their relatives [31]. Instead, results from our study highlight the need for further research on prevention of cardiovascular manifestations of Chagas’ disease which should be recognized in the agenda of health research priorities in endemic countries [44]. Although many endemic countries have implemented public health actions aimed to increase the use of benznidazole among children with chronic *T*. *cruzi* infection, treated children may still contribute to high direct healthcare costs because their higher risk for cardiovascular manifestations. Further longitudinal studies are needed to investigate whether tripanocides with new schemes can prevent cardiovascular outcomes beyond electrocardiographic abnormalities, including heart failure and sudden death, among children with chronic *T*. *cruzi* infection. This is important because there are few data available about the clinical significance of electrocardiographic abnormalities among children with chronic *T*. *cruzi* infection as most studies on heart conduction disturbances and Chagas disease have been conducted among adults [11, 13].

In conclusion, electrocardiographic abnormalities are common among children with chronic *T*. *cruzi* infection. There are several reasons for indicating trypanocidal therapy among children with chronic *T*. *cruzi* infection. However, results from the present study suggest that treatment with benznidazole for 60 days may not be associated with a lower occurrence of electrocardiographic abnormalities.

## Supporting Information

S1 Checklist STROBE checklist. (DOCX)

## References

[1] Pinto Dias JC. História natural da doença de Chagas. Arq Bras Cardiol. 1995;65(4):359–66. 8728813

[2] Silveira AC. O controle da doença de Chagas nos países do Cone Sul da América: história de uma iniciativa internacional, 1991/2001. Uberaba: Faculdade de Medicina do Triângulo Mineiro, Fundação de Ensino e Pesquisa de Uberaba; 2002. 316 p. p.

[3] Schmunis GA. The globalization of Chagas disease. ISBT Science Series. 2007;2(1):6–11. 10.1111/j.1751-2824.2007.00052.x

[4] World Health Organization, UNICEF/UNDP/World Bank/WHO Special Programme for Research and Training in Tropical Diseases, Pan American Health Organization. Reporte del grupo de trabajo científico sobre la enfermedad de Chagas: 17–20 de abril de 2005, actualizado en julio de 2007, Buenos Aires, Argentina. Geneva: World Health Organization; 2007. 96 p. p.

[5] Hotez PJ, Dumonteil E, Woc-Colburn L, Serpa JA, Bezek S, Edwards MS, et al Chagas disease: "the new HIV/AIDS of the Americas". PLoS Negl Trop Dis. 2012;6(5):e1498 Epub 2012/06/06. 10.1371/journal.pntd.0001498 22666504 PMC3362306

[6] Rassi A Jr., Rassi A, Marin-Neto JA. Chagas disease. Lancet. 2010;375(9723):1388–402. Epub 2010/04/20. 10.1016/s0140-6736(10)60061-x . 20399979

[7] Lee BY, Bacon KM, Bottazzi ME, Hotez PJ. Global economic burden of Chagas disease: a computational simulation model. Lancet Infect Dis. 2013;13(4):342–8. Epub 2013/02/12. 10.1016/s1473-3099(13)70002-1 23395248 PMC3763184

[8] Coura JR, Borges-Pereira J. Chronic phase of Chagas disease: why should it be treated? A comprehensive review. Mem Inst Oswaldo Cruz. 2011;106(6):641–5. doi: S0074-02762011000600001 [pii]. . 22012216 10.1590/s0074-02762011000600001

[9] Ribeiro AL, Nunes MP, Teixeira MM, Rocha MO. Diagnosis and management of Chagas disease and cardiomyopathy. Nat Rev Cardiol. 2012;9(10):576–89. Epub 2012/08/01. 10.1038/nrcardio.2012.109 . 22847166

[10] Salazar-Schettino PM, Perera R, Ruiz-Hernandez AL, Bucio Torres MI, Zamora-Gonzalez C, Cabrera-Bravo M, et al Chagas disease as a cause of symptomatic chronic myocardopathy in Mexican children. Pediatr Infect Dis J. 2009;28(11):1011–3. Epub 2009/10/28. 10.1097/INF.0b013e3181ad8425 . 19859016

[11] Viotti R, Vigliano C, Lococo B, Petti M, Bertocchi G, Alvarez MG, et al Clinical predictors of chronic chagasic myocarditis progression. Rev Esp Cardiol. 2005;58(9):1037–44. doi: 13078551 [pii]. . 16185616

[12] Sosa-Estani S, Viotti R, Segura EL. Therapy, diagnosis and prognosis of chronic Chagas disease: insight gained in Argentina. Mem Inst Oswaldo Cruz. 2009;104 Suppl 1:167–80. doi: S0074-02762009000900023 [pii]. . 19753472 10.1590/s0074-02762009000900023

[13] Viotti R, Vigliano C, Armenti H, Segura E. Treatment of chronic Chagas' disease with benznidazole: clinical and serologic evolution of patients with long-term follow-up. Am Heart J. 1994;127(1):151–62. Epub 1994/01/01. . 8273735 10.1016/0002-8703(94)90521-5

[14] de Andrade AL, Zicker F, de Oliveira RM, Almeida Silva S, Luquetti A, Travassos LR, et al Randomised trial of efficacy of benznidazole in treatment of early *Trypanosoma cruzi* infection. Lancet. 1996;348(9039):1407–13. Epub 1996/11/23. . 8937280 10.1016/s0140-6736(96)04128-1

[15] Sosa Estani S, Segura EL, Ruiz AM, Velazquez E, Porcel BM, Yampotis C. Efficacy of chemotherapy with benznidazole in children in the indeterminate phase of Chagas' disease. Am J Trop Med Hyg. 1998;59(4):526–9. . 9790423 10.4269/ajtmh.1998.59.526

[16] Villar JC, Perez JG, Cortes OL, Riarte A, Pepper M, Marin-Neto JA, et al Trypanocidal drugs for chronic asymptomatic *Trypanosoma cruzi* infection. Cochrane Database Syst Rev. 2014;5:CD003463 Epub 2014/05/29. 10.1002/14651858.CD003463.pub2 . 24867876 PMC7154579

[17] Molina I, Gomez i Prat J, Salvador F, Trevino B, Sulleiro E, Serre N, et al Randomized trial of posaconazole and benznidazole for chronic Chagas' disease. N Engl J Med. 2014;370(20):1899–908. Epub 2014/05/16. 10.1056/NEJMoa1313122 . 24827034

[18] Andrade JP, Marin-Neto JA, Paola AA, Vilas-Boas F, Oliveira GM, Bacal F, et al I Latin American Guidelines for the diagnosis and treatment of Chagas' heart disease. Arq Bras Cardiol. 2011;97(2 Suppl 3):1–48. Epub 2011/10/14. . 21952638

[19] Bern C. Antitrypanosomal therapy for chronic Chagas' disease. N Engl J Med. 2011;364(26):2527–34. 10.1056/NEJMct1014204 . 21714649

[20] Garcia S, Ramos CO, Senra JF, Vilas-Boas F, Rodrigues MM, Campos-de-Carvalho AC, et al Treatment with benznidazole during the chronic phase of experimental Chagas' disease decreases cardiac alterations. Antimicrob Agents Chemother. 2005;49(4):1521–8. Epub 2005/03/29. 10.1128/aac.49.4.1521–1528.2005 15793134 PMC1068607

[21] Morillo CA, Marin-Neto JA, Avezum A, Sosa-Estani S, Rassi A Jr., Rosas F, et al Randomized Trial of Benznidazole for Chronic Chagas' Cardiomyopathy. N Engl J Med. 2015;373(14):1295–306. Epub 2015/09/02. 10.1056/NEJMoa1507574 . 26323937

[22] Bern C. Chagas' Disease. N Engl J Med. 2015;373(5):456–66. Epub 2015/07/30. 10.1056/NEJMra1410150 . 26222561

[23] Lazzari JO, Pereira M, Antunes CM, Guimaraes A, Moncayo A, Chavez Dominguez R, et al Diagnostic electrocardiography in epidemiological studies of Chagas' disease: multicenter evaluation of a standardized method. Rev Panam Salud Publica. 1998;4(5):317–30. doi: S1020-49891998001100005 [pii]. . 9883073 10.1590/s1020-49891998001100005

[24] VanderWeele TJ. On the distinction between interaction and effect modification. Epidemiology. 2009;20(6):863–71. Epub 2009/10/07. 10.1097/EDE.0b013e3181ba333c . 19806059

[25] Viotti R, Vigliano C, Lococo B, Bertocchi G, Petti M, Alvarez MG, et al Long-term cardiac outcomes of treating chronic Chagas disease with benznidazole versus no treatment: a nonrandomized trial. Ann Intern Med. 2006;144(10):724–34. doi: 144/10/724 [pii]. . 16702588 10.7326/0003-4819-144-10-200605160-00006

[26] Coura JR, de Abreu LL, Willcox HP, Petana W. Estudo comparativo controlado com emprego de benznidazole, nifurtimox e placebo, na forma crônica da doença de Chagas, em uma área de campo com transmissão interrompida. I. Avaliação preliminar. Rev Soc Bras Med Trop. 1997;30(2):139–44. . 9148337 10.1590/s0037-86821997000200009

[27] Singh RS, Totawattage DP. The Statistical Analysis of Interval-Censored Failure Time Data with Applications. Open J Stat. 2013;3:155–66.

[28] Collett D. Modelling survival data in medical research 2nd ed. Boca Raton, FL: Chapman & Hall/CRC; 2003. 178–9 p.

[29] Goncalves JG, Prata A. Estudo comparativo de três códigos para leitura de eletrocardiogramas na doença de Chagas crônica. Rev Panam Salud Publica. 2003;14(3):201–8. doi: S1020-49892003000800007 [pii]. . 14653907 10.1590/s1020-49892003000800007

[30] Guedes PM, Silva GK, Gutierrez FR, Silva JS. Current status of Chagas disease chemotherapy. Expert Rev Anti Infect Ther. 2011;9(5):609–20. Epub 2011/05/26. 10.1586/eri.11.31 . 21609270

[31] Sosa-Estani S, Colantonio L, Segura EL. Therapy of chagas disease: implications for levels of prevention. J Trop Med. 2012;2012:292138 10.1155/2012/292138 22523499 PMC3317183

[32] Andrade AL, Martelli CM, Oliveira RM, Silva SA, Aires AI, Soussumi LM, et al Short report: benznidazole efficacy among *Trypanosoma cruzi*-infected adolescents after a six-year follow-up. Am J Trop Med Hyg. 2004;71(5):594–7. Epub 2004/12/01. . 15569790

[33] Viotti R, Alarcon de Noya B, Araujo-Jorge T, Grijalva MJ, Guhl F, Lopez MC, et al Towards a paradigm shift in the treatment of chronic Chagas disease. Antimicrob Agents Chemother. 2014;58(2):635–9. Epub 2013/11/20. 10.1128/aac.01662-13 24247135 PMC3910900

[34] Perez-Molina JA, Perez-Ayala A, Moreno S, Fernandez-Gonzalez MC, Zamora J, Lopez-Velez R. Use of benznidazole to treat chronic Chagas' disease: a systematic review with a meta-analysis. J Antimicrob Chemother. 2009;64(6):1139–47. Epub 2009/10/13. 10.1093/jac/dkp357 . 19819909

[35] Guías para la atención al paciente infectado con *Trypanosoma cruzi* (Enfermedad de Chagas) Buenos Aires, Argentina: Ministerio de Salud de la Nación; 2012. Available from: http://www.msal.gob.ar/images/stories/bes/graficos/0000000622cnt-03-guia-para-la-atencion-al-paciente-con-chagas.pdf.

[36] Riarte A. TRAENA–placebo-controlled evaluation of impact of benznidazole treatment on long term disease progression in adults with chronic Chagas disease (abstract). American Society of Tropical Medicine and Hygiene (ASTMH), 62nd Annual Meeting. Washington, DC, USA: ASTMH; 2013.

[37] Sguassero Y, Cuesta CB, Roberts KN, Hicks E, Comande D, Ciapponi A, et al Course of Chronic *Trypanosoma cruzi* Infection after Treatment Based on Parasitological and Serological Tests: A Systematic Review of Follow-Up Studies. PLoS One. 2015;10(10):e0139363 Epub 2015/10/06. 10.1371/journal.pone.0139363 26436678 PMC4593559

[38] Sosa-Estani S, Segura EL. Etiological treatment in patients infected by *Trypanosoma cruzi*: experiences in Argentina. Curr Opin Infect Dis. 2006;19(6):583–7. 10.1097/01.qco.0000247592.21295.a5 00001432-200612000-00010 [pii]. . 17075335

[39] Kierszenbaum F. Where do we stand on the autoimmunity hypothesis of Chagas disease? Trends Parasitol. 2005;21(11):513–6. Epub 2005/08/30. 10.1016/j.pt.2005.08.013 . 16125464

[40] Bussink BE, Holst AG, Jespersen L, Deckers JW, Jensen GB, Prescott E. Right bundle branch block: prevalence, risk factors, and outcome in the general population: results from the Copenhagen City Heart Study. Eur Heart J. 2013;34(2):138–46. Epub 2012/09/06. 10.1093/eurheartj/ehs291 . 22947613

[41] Sosa-Estani S, Cura E, Velazquez E, Yampotis C, Segura EL. Etiological treatment of young women infected with *Trypanosoma cruzi*, and prevention of congenital transmission. Rev Soc Bras Med Trop. 2009;42(5):484–7. Epub 2009/12/08. . 19967227 10.1590/s0037-86822009000500002

[42] Fabbro DL, Danesi E, Olivera V, Codebo MO, Denner S, Heredia C, et al Trypanocide treatment of women infected with *Trypanosoma cruzi* and its effect on preventing congenital Chagas. PLoS Negl Trop Dis. 2014;8(11):e3312 Epub 2014/11/21. 10.1371/journal.pntd.0003312 25411847 PMC4239005

[43] Moscatelli G, Moroni S, Garcia-Bournissen F, Ballering G, Bisio M, Freilij H, et al Prevention of congenital Chagas through treatment of girls and women of childbearing age. Mem Inst Oswaldo Cruz. 2015;110(4):507–9. Epub 2015/05/21. 10.1590/0074-02760140347 25993401 PMC4501414

[44] Colantonio LD, Baldridge AS, Huffman MD, Bloomfield GS, Prabhakaran D. Cardiovascular Research Publications from Latin America between 1999 and 2008. A Bibliometric Study. Arq Bras Cardiol. 2015;104(1):5–14. Epub 2015/02/26. 10.5935/abc.20140213 . 25714407 PMC4387606

