# Supplementary material for: Electrocardiographic Abnormalities and Treatment with Benznidazole among Children with Chronic Infection by Trypanosoma cruzi: A Retrospective Cohort Study
Source: PLoS Negl Trop Dis. 2016 May 9;10(5):e0004651. doi: 10.1371/journal.pntd.0004651 (PMC4861278; doi:10.1371/journal.pntd.0004651)
Supplement: S1 Checklist — (DOCX) [file pntd.0004651.s001.docx]

STROBE Statement—checklist of items that should be included in reports of observational studies

|  | Item No. | Recommendation | Page  No. | Relevant text from manuscript |
| --- | --- | --- | --- | --- |
| **Title and abstract** | 1 | (*a*) Indicate the study’s design with a commonly used term in the title or the abstract | 1 | - A retrospective cohort study. |
|  |  | (*b*) Provide in the abstract an informative and balanced summary of what was done and what was found | 3 | - We sought to analyze electrocardiographic abnormalities among children with chronic T. cruzi infection with and without trypanocidal treatment with benznidazole. - Multivariable adjusted prevalence ratios (95% confidence interval [95%CI]) for electrocardiographic abnormalities in 1995-1996, 1998, 2000 and 2005 comparing children treated with benznidazole versus those not treated were 2.76 (0.66, 11.60), 2.33 (0.44, 12.31), 3.06 (0.48, 19.56), and 1.94 (0.33, 11.25), respectively. |
| Introduction | | | |  |
| Background/rationale | 2 | Explain the scientific background and rationale for the investigation being reported | 6-7 | - Evidence from animal models suggest that treatment with benznidazole could prevent or control chagasic cardiomyopathy [20], although results from observational studies have been controversial [16]. - Few studies analyzed the characteristics and natural history of electrocardiographic abnormalities among children with chronic T. cruzi infection and the effect associated with treatment with benznidazole [16, 19]. |
| Objectives | 3 | State specific objectives, including any prespecified hypotheses | 7 | - The main objective of the present study was to investigate the presence of electrocardiographic abnormalities in a cohort of children with chronic T. cruzi infection, some of whom received treatment with benznidazole. We hypothesized that electrocardiographic abnormalities will be frequent among children with chronic T. cruzi infection and less common among those treated with benznidazole versus those not treated. |
| Methods | | | |  |
| Study design | 4 | Present key elements of study design early in the paper | 7 | - We conducted a retrospective cohort study using data collected during a double-blind randomized controlled clinical trial with extended follow-up. |
| Setting | 5 | Describe the setting, locations, and relevant dates, including periods of recruitment, exposure, follow-up, and data collection | 7-9 | - The clinical trial was conducted in Salta province, Argentina from 1991 through 1996. - The region where the study was conducted had continuous surveillance for T. cruzi vectors by sanitary agents since 1982, and the possibility of reinfection after treatment was considered low. - During enrolment in 1991-1992, - Children included.in the clinical trial were matched by age and place of residence, and randomly assigned to benznidazole 5 mg/Kg/day (benznidazole group, n=55) or placebo (placebo group, n=51) for 60 days. - At the end of the clinical trial, all participants in the placebo group were offered treatment with benznidazole if follow-up for adverse events was considered feasible. A total of 18 children in the placebo group completed a properly documented treatment with benznidazole open-label in 1997. - A cohort of 19 children with asymptomatic chronic infection by T. cruzi who completed the study anamnesis, physical examination and serology tests but were not included in the clinical trial received treated with benznidazol open-label 5 mg/Kg/day for 60 days in 1991-1992 (benznidazole cohort). - Electrocardiograms were obtained from children enrolled in the clinical trial and in the benznidazole cohort in 1991-1992, 1995-1996, 1998, 2000 and 2005. |
| Participants | 6 | (*a*) *Cohort study*—Give the eligibility criteria, and the sources and methods of selection of participants. Describe methods of follow-up  *Case-control study*—Give the eligibility criteria, and the sources and methods of case ascertainment and control selection. Give the rationale for the choice of cases and controls  *Cross-sectional study*—Give the eligibility criteria, and the sources and methods of selection of participants | 9 | - Electrocardiograms were obtained from children enrolled in the clinical trial and in the benznidazole cohort in 1991-1992, 1995-1996, 1998, 2000 and 2005. For the present analysis, we included children enrolled in the clinical trial or in the benznidazole cohort who had at least 1 valid electrocardiogram to assess electrocardiographic abnormalities. |
|  |  | (*b*) *Cohort study*—For matched studies, give matching criteria and number of exposed and unexposed  *Case-control study*—For matched studies, give matching criteria and the number of controls per case | 8 | - Children included in the clinical trial were matched by age and place of residence, and randomly assigned to benznidazole 5 mg/Kg/day (benznidazole group, n=55) or placebo (placebo group, n=51) for 60 days. |
| Variables | 7 | Clearly define all outcomes, exposures, predictors, potential confounders, and effect modifiers. Give diagnostic criteria, if applicable | 10, 12 | Outcome. Page 10:   - Electrocardiographic abnormalities were defined by any abnormal finding identified using the Buenos Aires method.   Exposure. Page 12:   - For our main analysis, we considered that children randomly assigned to benznidazole in the clinical trial and those in the benznidazole cohort were treated with this medication (intention-to-treat analysis).   Confounders. Page 10:   - Potential confounders assessed at baseline which were used for statistical adjustment in the present analysis include age, gender, body weight and rural residence. |
| Data sources/ measurement | 8* | For each variable of interest, give sources of data and details of methods of assessment (measurement). Describe comparability of assessment methods if there is more than one group | 8-10 | Page 8:   - During enrolment in 1991-1992, children attending local elementary schools were screened by history, physical examination and 3 serology tests for T. cruzi using different techniques: indirect hemagglutination inhibition, indirect immunofluorescence assay, and enzyme-linked immunosorbent assay. Of relevance to the current analysis, information on age, sex, body weight and place of residence was collected.   Page 9:   - Electrocardiograms were obtained from children enrolled in the clinical trial and in the benznidazole cohort in 1991-1992, 1995-1996, 1998, 2000 and 2005.   Page 10:   - All electrocardiograms were analyzed by the same cardiologist (NP) using the Buenos Aires method [23]. |
| Bias | 9 | Describe any efforts to address potential sources of bias | 1-13 | - Children included in the clinical trial were matched by age and place of residence, and randomly assigned to benznidazole 5 mg/Kg/day (benznidazole group, n=55) or placebo (placebo group, n=51) for 60 days. - In addition to a crude model, a multivariable adjusted model was fit, including adjustment for age, gender, body weight and rural residence. |
| Study size | 10 | Explain how the study size was arrived at | 9 | This is a retrospective cohort study. Therefore, the sample size was not defined a priori but a consequence of data availability and the inclusion criteria.  Page 9:   - For the present analysis, we included children enrolled in the clinical trial or in the benznidazole cohort who had at least 1 valid electrocardiogram to assess electrocardiographic abnormalities.   Page 13:   - Between 1991 and 2005, 500 electrocardiograms were obtained from this population. We excluded electrocardiograms with missing date (n=1) and those coded as not evaluable by the cardiologist (n=14). After these exclusions, 111 children had at least 1 valid electrocardiogram during the study period (485 electrocardiograms in total) and were included in the current analysis. |

Continued on next page

| Quantitative variables | 11 | Explain how quantitative variables were handled in the analyses. If applicable, describe which groupings were chosen and why | 10-13 | - Baseline characteristics of participants included in the current analysis who were randomly assigned to benznidazole and placebo, separately, and those in the benznidazole cohort are reported using median and 25th-75th percentiles for continuous variables, and percentage for binary variables. Differences between groups were analyzed using Kruskal-Wallis or Fisher’s exact tests, as appropriated. |
| --- | --- | --- | --- | --- |
| Statistical methods | 12 | (*a*) Describe all statistical methods, including those used to control for confounding | 10-13 | All statistical analyses are fully described in pages 10-13 |
|  |  | (*b*) Describe any methods used to examine subgroups and interactions | 12 | - We conducted analyses limited to children without electrocardiographic abnormalities at baseline. |
|  |  | (*c*) Explain how missing data were addressed | 9, 11-12 | Page 9:   - For the present analysis, we included children enrolled in the clinical trial or in the benznidazole cohort who had at least 1 valid electrocardiogram to assess electrocardiographic abnormalities.   Page 11:   - We used the last observation carried forward method for the main analysis because some participants did not have an electrocardiogram in each assessment period.   Page 12:   - Several sensitivity analyses of the association between treatment with benznidazole and electrocardiographic abnormalities were conducted. First, we repeated the analysis without using the last observation carried forward method. |
|  |  | (*d*) *Cohort study*—If applicable, explain how loss to follow-up was addressed  *Case-control study*—If applicable, explain how matching of cases and controls was addressed  *Cross-sectional study*—If applicable, describe analytical methods taking account of sampling strategy | 11-12 | Page 11:   - We used the last observation carried forward method for the main analysis because some participants did not have an electrocardiogram in each assessment period.   Page 12:  Several sensitivity analyses of the association between treatment with benznidazole and electrocardiographic abnormalities were conducted. First, we repeated the analysis without using the last observation carried forward method. |
|  |  | (*e*) Describe any sensitivity analyses | 12 | - Several sensitivity analyses of the association between treatment with benznidazole and electrocardiographic abnormalities were conducted. First, we repeated the analysis without using the last observation carried forward method. Second, we conducted a per-protocol analysis, considering children who did not complete 30 days of treatment with benznidazole as not treated, and children who received treatment with benznidazole in 1997 as treated in 1998, 2000 and 2005. We used 30 days to determine whether children were treated as prior studies have shown that a treatment with benznidazole shorter than 60 days can be effective to induce T. cruzi clearance [25, 26]. Finally, we repeated the analysis limited to children enrolled in the randomized controlled clinical trial. |
| Results | | | | |
| Participants | 13* | (a) Report numbers of individuals at each stage of study—eg numbers potentially eligible, examined for eligibility, confirmed eligible, included in the study, completing follow-up, and analysed | 15 | Numbers of individuals at each stage of study are reported in Figure 1. |
|  |  | (b) Give reasons for non-participation at each stage | 14 | - The number of children who participated at follow-up examinations and had an electrocardiogram was reduced over time, mainly because migration. |
|  |  | (c) Consider use of a flow diagram | 15 | A flowchart is presented in Figure 1 |
| Descriptive data | 14* | (a) Give characteristics of study participants (eg demographic, clinical, social) and information on exposures and potential confounders | 15 | Characteristics of study participants are reported in Table 1 |
|  |  | (b) Indicate number of participants with missing data for each variable of interest | 15 | Footnote of Table 1:   - Note: Variables reported have no missing data. |
|  |  | (c) *Cohort study*—Summarise follow-up time (eg, average and total amount) | 14 | Median follow-up (25th-75th percentile) was 8.6 (7.1-14.1) years. |
| Outcome data | 15* | *Cohort study*—Report numbers of outcome events or summary measures over time | 16, 21 | Page 16:   - A total of 94 children had an electrocardiogram in 1991-1992, including 8 (8.5%) children with electrocardiographic abnormalities.   Page 21:   - A total of 16 (18.6%) children developed incident electrocardiographic abnormalities during follow-up. |
|  |  | *Case-control study—*Report numbers in each exposure category, or summary measures of exposure | NA | NA |
|  |  | *Cross-sectional study—*Report numbers of outcome events or summary measures | NA | NA |
| Main results | 16 | (*a*) Give unadjusted estimates and, if applicable, confounder-adjusted estimates and their precision (eg, 95% confidence interval). Make clear which confounders were adjusted for and why they were included | 20 | Estimates and 95% confidence intervals are provided in Table 3. |
|  |  | (*b*) Report category boundaries when continuous variables were categorized | NA | NA |
|  |  | (*c*) If relevant, consider translating estimates of relative risk into absolute risk for a meaningful time period | NA | NA |

Continued on next page

| Other analyses | 17 | Report other analyses done—eg analyses of subgroups and interactions, and sensitivity analyses | 20-21 | Results from sensitivity analyses are reported in Table 3. Results for the analysis limited to participants without electrocardiographic abnormalities at baseline is reported in Page 21:   - The crude hazard ratio for incident electrocardiographic abnormalities comparing children treated with benznidazole versus those not treated was 0.74 (95% CI: 0.28-1.97, p-value: 0.54). After adjustment for age at baseline, gender, rural residence and body weight, the hazard ratio for incident electrocardiographic abnormalities associated with treatment with benznidazole was 0.68 (95% CI: 0.25-1.88, p-value: 0.46). |
| --- | --- | --- | --- | --- |
| Discussion | | | | |
| Key results | 18 | Summarise key results with reference to study objectives | 23 | - In our analysis, children with chronic T. cruzi infection frequently presented or developed electrocardiographic abnormalities. After statistical adjustment, treatment with benznidazole for 60 days was not associated with less electrocardiographic abnormalities as compared with no treatment over a median follow-up of 8.6 years. |
| Limitations | 19 | Discuss limitations of the study, taking into account sources of potential bias or imprecision. Discuss both direction and magnitude of any potential bias | 26 | - Several factors may have contributed to attenuate a possible association between treatment with benznidazole and lower risk for electrocardiographic abnormalities in our study. Some children who received treatment with benznidazole may have remained with persistent T. cruzi infection. We cannot exclude the possibility of reinfection among children successfully treated with benznidazole, although this was considered unlikely. Also, some children who were analyzed as untreated in the present study may have received treatment with benznidazole during follow-up, which was not documented. Some electrocardiographic abnormalities observed in this analysis, including bundle branch block, may be unrelated with T. cruzi infection as they may also be detected among uninfected children [29, 40]. |
| Interpretation | 20 | Give a cautious overall interpretation of results considering objectives, limitations, multiplicity of analyses, results from similar studies, and other relevant evidence | 28 | - Results from the present study suggest that treatment with benznidazole for 60 days may not be associated with a lower occurrence of electrocardiographic abnormalities. |
| Generalisability | 21 | Discuss the generalisability (external validity) of the study results | 27 | - Finally, because the study was conducted in a restricted geographic area, results may not be generalizable to children with chronic T. cruzi infection from other regions. |
| Other information | |  | | |
| Funding | 22 | Give the source of funding and the role of the funders for the present study and, if applicable, for the original study on which the present article is based | 2 | - This study was supported by the Training and Research on Tropical Disease (TDR) UPND/WB/WHO/TDR Program, the National Ministry of Health of Argentina, and the Ministry of Health of the Province of Salta, Argentina. The funders had no role in study design, data collection and analysis, decision to publish, or preparation of the manuscript. Dr. Colantonio was supported by a Fulbright scholarship to complete the PhD program in Epidemiology from the University of Alabama at Birmingham. |

*Give information separately for cases and controls in case-control studies and, if applicable, for exposed and unexposed groups in cohort and cross-sectional studies.

**Note:** An Explanation and Elaboration article discusses each checklist item and gives methodological background and published examples of transparent reporting. The STROBE checklist is best used in conjunction with this article (freely available on the Web sites of PLoS Medicine at http://www.plosmedicine.org/, Annals of Internal Medicine at http://www.annals.org/, and Epidemiology at http://www.epidem.com/). Information on the STROBE Initiative is available at www.strobe-statement.org.
